# Measurement Scales of Suicidal Ideation and Attitudes: A Systematic Review Article

**DOI:** 10.15171/hpp.2015.019

**Published:** 2015-10-25

**Authors:** Parvin Ghasemi, Abdolreza Shaghaghi, Hamid Allahverdipour

**Affiliations:** ^1^Health Education and Promotion Department, Tabriz University of Medical Sciences, Tabriz, Iran; ^2^Medical Education Research Center, Tabriz University of Medical Sciences, Tabriz, Iran; ^3^Research Center of Psychiatry and Behavioral Sciences, Tabriz University of Medical Sciences, Tabriz, Iran

**Keywords:** Auicide, Suicidal Ideation, Attitude, Scale, Iran

## Abstract

**Background:** The main aim of this study was to accumulate research evidence that introduce validated scales to measure suicidal attitudes and ideation and provide an empirical framework for adopting a relevant assessment tool in studies on suicide and suicidal behaviors.

**Methods:** Medical Subject Headings’ (MeSH) terms were used to search Ovid Medline, PROQUEST, Wiley online library, Science Direct and PubMed for the published articles in English that reported application of an scale to measure suicidal attitudes and ideation from January 1974 onward.

**Results:** Fourteen suicidal attitude scale and 15 scales for assessing suicidal ideation were identified in this systematic review. No gold standard approach was recognized to study suicide related attitudes and ideations.

**Conclusion:** Special focus on generally agreed dimensions of suicidal ideation and attitudes and cross-cultural validation of the introduced scales to be applicable in different ethnic and socially diverse populations could be a promising area of research for scholars.

## Introduction

Suicide is a challenging public health dilemma worldwide^1-3^ and according to WHO estimates, approximately 1 million people are victims of suicide each year.^4^ Considering the extent of under reporting of the suicide cases due to the causes such as lack of robust registration systems or stigma against suicide attempters and their families, the real number of suicide seems to be even higher so that it is regarded as one of the prominent causes of death especially among people of young ages in the world.^1,5,6^

Physical, mental and economic burden of suicide on families and larger societies could be overwhelming and its prevalence rate could be an important index of communities’ health.^1^ Therefore, studies that are focusing on the understanding and prevention of suicidal behaviors might have a high priority in the list of countries’ health priorities.^7^

Complex and multifaceted nature of pathways that lead to suicidal behavior will make application of an explicit research methodology inevitable in studies on this disturbing phenomenon.^3,8^Infinite understanding of the social and psychological factors that may prone an individual to suicide could also help program planners in amelioration of the prevention strategies.^9^

Suicidal attitudes and ideation are key antecedents in studying pathways and mechanisms that could lead to suicide.^10,11^It is estimated that within the general populations, 2.1-18.5% of people had seriously considered committing suicide in their life span.^10,12^Estimates of the lifetime incidence of suicide attempts also range from 0.7% to 5.9% depending on the demographic characteristics of the group being sampled.^10,12^

Cross culturally validated scales could be employed to study attitudes toward suicide in different countries based on assumption that suicidal thoughts and attitudes share common pattern in different communities and only type of suicide, seriousness of suicide attempts and its scale may differ based on the cultural milieu.^11,12^

Kodaka et al.^13^ in their research to identify attitudinal scales to be applicable in studies on attitudes toward suicide have reported three valid and reliable scales. Hourani at al.^14^ have investigated suicide assessment methodology with special focus on suicide specific instruments developed between 1966 and 1999. Psychometric properties of different scales to assess suicidal ideation and behavior were also inspected earlier.^7,15,16^Our overall judgment however, is lack of cumulative update evidence to help researchers in studies to speculate suicidal attitude and ideation.

The main purpose of this study was to accumulate research evidence that introduce validated scales to measure suicidal attitudes and ideation and provide an empirical framework for adopting a relevant assessment tool in studies on suicide and suicidal behaviors.

## Materials and Methods

The terms suicide AND attitude AND scale OR assessment OR questionnaire, and also suicide AND ideation AND scale OR assessment OR questionnaire were used to investigate Ovid Medline, PROQUEST, Wiley online library, Science Direct and PubMed for the relevant published articles in English that reported application of an scale to measure suicidal attitudes and ideation from 1974 onward. The search was conducted from inception to November 2013 and at the first stage, titles and abstracts of retrieved publications were screened and non-relevant reports excluded. Full texts of remaining publications were acquired and thoroughly inspected for inclusion if adhered to the predetermined inclusion criteria.

Super searcher of Google Scholar was also checked for grey literature in the purposed time limit. Quality of the studies was determined based on the proven validity of the introduced scale and report of the applied scale’s specifications. All authors independently assessed the identified publications and any disagreement regarding the eligibility of a paper for inclusion was resolved with consensus. A customized data extraction sheet was used to espouse the relevant data about assessment tools of attitude toward suicide and suicide ideation.

### Ethical Considerations

All efforts have been made to avoid redundant publications and provide maximal possible accuracy in presenting the findings. Required details to ensure application of a sensitive search strategy were also acquainted to make the applied method as transparent as that might be assumed.

## Results

**Fig. 1:**
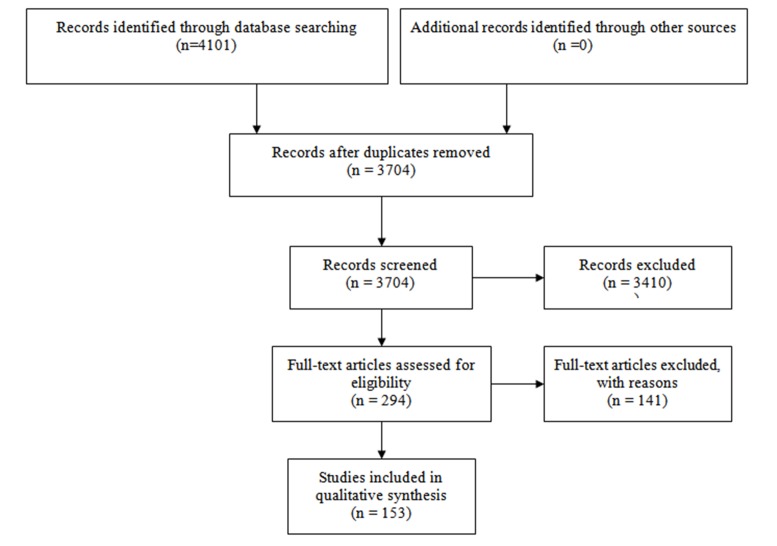
PRISMA flow diagram to detail study search findings

A total of 4101 articles were initially identified through the search strategy. Among the identified reports, 397 duplicates were excluded. After screening the titles and abstracts of the remaining 3704 records, 3012 publications that did not meet the inclusion criteria were also precluded. Lack of access to the full text of 398 articles was also made their exclusion inevitable. Finally, full texts of 294 eligible articles were scrutinized to identify measurement scales of suicidal attitudes and ideation and only 153 had the inclusion criteria (Figure 1). Of the remaining publications, 14 instruments to assess suicidal attitudes and 21 scales to study suicidal ideation were pinpointed.

The recognized measures were included a variety of tools to study suicidal and other closely related behaviors associated with suicide risk. These scales pertain to the following categories of assessment measures: suicide ideation and behavior, lethality of suicide attempts, reasoning mechanisms of suicide attempters and health care providers’ attitudes towards suicide attempt survivors.

The retrieved scales’ name, their introducers’ name, year of the introduction, number of items within each individual scale and a summary of their specifications were tabulated in Table 1 and Table 2.

Brief descriptions about the identified tools are provided as follows:

### Suicidal attitudes scales

1.Suicide Opinion Questionnaire (SOQ): The questionnaire contains 100 items that ask respondents attitude in eight domains.^11,12-17^

- Mental illness (suicide reflects mental illness);

- Cry for help (suicide threats are not real, they represent a cry for help);

-Right to die (people have the right to take their own lives);

-Religion (lack of religion has a role in suicide);

-Impulsivity (deliberate self-harm and suicide are impulsive acts) ;

-Normality (everyone is potentially capable of suicide);

-Aggression (suicide is an aggressive act), and Moral evil (suicide is a morally bad action)

2.Multi-Attitude Suicide Tendency Scale (MAST):

The MAST is a 30-item self-report measure designed to evaluate conflicting attitudes related to life and death. The domains of attitudes assessed with the MAST include attraction to life, repulsion by life, attraction to death, and repulsion by death.^5,18-20^

The MAST-II is a revised 24-item self-report instrument, reframed to include the same general constructs as with the original MAST. The MAST-II differs from other suicide assessment methods as it includes both risk and protective factors.^5^

**Table 1:** Main characteristics of the identified suicidal attitudes scales

**Scale name**	**Year of the publication**	**Description & purpose**	**Items**	**Administration**		**Target group**					
				**Self-Report**	**Interview**	**Psych. patients**	**College students**	**Adolescents**	**Adults**	**Community-based**	**Other**
Suicide Opinion Questionnaire, Domino et al. (SOQ)	1982	Attitudes towards suicide	100	*			*	*	*	*	*
Suicide Attitude Vignette Experience, Stillion. (SAVE)	1984	The acquisition of attitudes towards suicidal behaviors.	16		*		*	*	*		*
Suicide Attitude Questionnaire, Diekstra. (SUIATT)	1985	Perceptions on suicide, circumstances leading to suicidal ideation.	63	*			*			*	*
Multi-Attitude Suicide Tendency Scale, Orbach et al. (MAST)	1991	Attitude toward life and death.	30	*		*	*	*	*		
General Social Survey's, Davis & Smith. (GSS 4)	1993	Questions about suicidal attitudes are justifiable in the life crises.	4	*						*	
Semantic Differential Scale Attitudes towards Suicidal Behavior, Jenner et al. (SEDAS)	2001	Attitudes towards suicidal behavior of different actors in various situations.	15		*					*	
Suicide Attitudes and Attribution Scale, Sorjonen (SAAS).	2002	Suicidal act and suicides’ character.	36	*			*				*
Attitudes Toward Suicide Scale,Renberg &amp;amp;amp; Jacobsson (ATTS).	2003	Attitudes toward suicide in the general population.	61	*			*		*	*	
Attitude Towards Suicide Scale, Eskin's. (ATSS)	2004	Right to commit suicide or whether suicide a sign is mental illness, also refers to social aspects, such as communication around the topic of suicide.	24	*				*			*
Attitudinal Beliefs Towards Suicidal Behavior Scale, Ruiz Hernández et al. (CCCS-18)	2005	Attitudes towards suicide has the scale, as a supplement to other instruments	18		*			*			*
Suicide Behavior Attitude Questionnaire, Botega,et al. (SBAQ)	2005	Attitude towards suicide among nursing personnel which measures attitudes of nursing personnel towards suicide.	21	*						*	*
Hong Kong version of the Chinese Attitude toward Suicide Questionnaire, Sing Lee et al. (CASQ-HK)	2007	Attitudes toward suicide and prior suicidal experience.	73	*						*	
Attitudes Towards Attempted Suicide Questionnaire, Ouzouni &amp;amp;amp; Nakakis. (ATAS-Q)	2009	Attitudes health care professional towards people who have attempted suicide.	80	*							*
Scale of public attitudes about suicide, Li XY et al. (SPAS)	2011	To assess knowledge about suicide and seven specific attitudes about suicide	47	*		*	*			*	*

**Table 2:** Main characteristics of the identified suicidal ideation scales

**Scale name**	**Year of the publication**	**Description &amp; purpose**	**Items**	**Administration**		**Target group**					
				**Self-Report**	**Interview**	**Psych. patients**	**Medical**	**College students**	**Adolescents**	**Adults**	**Community-based**
Paykel’s questionnaire, Paykel et al.	1974	Suicidal thoughts and attempts.	5	*				*	*	*	*
Modified Scale for Suicidal Ideation, Miller et al. (MSSI)	1986	Suicide Ideation.	18		*			*	*	*	
Suicidal Ideation Questionnaire, Reynolds. (SIQ)	1988	Specific thoughts and cognitions about suicide and death.	30	*		*			*	*	
The Suicidal Ideation Scale, Rudd. (SIS)	1989	Severity or intensity of suicidal ideation.	10	*		*		*			
Adult Suicidal Ideation Questionnaire, Reynolds. (ASIQ)	1991	Current level of suicidal ideation.	25	*		*		*		*	
Beck Scale for Suicide Ideation, Beck & Steer. (BSSI)	1991	Suicide Ideation.	21	*		*		*	*	*	
Suicidal Ideation Screening Questionnaire, Cooper Patrick. (SIS-Q)	1994	Sleep disturbance, mood disturbance, guilt and hopelessness.	4		*					*	
Suicide Probability Scale, Cull and Gill. (SPS)	1995	Attitudes/behaviors related to suicide risk and suicidal ideation.	36	*		*		*	*	*	*
Suicidal Behaviors Questionnaire, Linehan. (SBQ)	1996	Measure of suicidal thoughts and past attempts.	34	*		*			*	*	
Positive and Negative Suicide Ideation, Osman, et al.(PANSI)	1998	The frequency of protective and negative risk dimensions of suicidal ideation.	10	*		*		*	*		
Suicidal Behaviors Questionnaire – Revised, Osman et al. (SBQ-R)	2001	Self-report measure of suicidal thoughts and past attempts.	4	*		*			*		
InterSePT Scale for Suicidal Thinking, Lindenmayer et al. (ISST)	2003	Suicidal ideation in patients with schizophrenia and schizoaffective disorders.	12	*		*					
Geriatric suicide ideation scale, Heisel et al. (GSIS)	2006	Suicide ideation and related factors.	31	*	*						
Columbia Suicide Severity Rating Scale, Posner et al. (C-SSRS).	2008	Suicide-related ideation and behavior and intensity of the ideation.	23		*	*			*	*	
Brief Symptom Rating Scale, Lung et al. (BSRS-5)	2008	Suicide ideation.	5	*		*				*	*

3.Attitudes Toward Suicide Scale (ATTS):

The ATTS scale consists of 61 items. It was developed based on the Suicide Opinion Questionnaire (SOQ). The questionnaire contains five sections^1,11,21-25^ that include items to collect data about:

- Related experience of suicidal problems among significant others,

-Attitudes towards suicide (the main part) including broad dimensions such as attitudes towards suicide as a right, incomprehensibility of suicide, non-communicability of suicide, its preventability, consideration of suicide as a taboo or as a normal common act, attitudes toward suicide process, consideration of suicide as a relation–caused phenomenon and preparedness to prevent and resignation,

- Life satisfaction and suicidal expressions,

- Related demographic data,

- Suicide causes and means of prevention.

4.Attitudes Towards Attempted Suicide Questionnaire (ATAS-Q):

The ATAS-Q that comprises 80 attitudinal items is a useful tool in measuring attitudes of people who have attempted suicide. This scale may help health care professionals to enhance their understanding about attitudes of patients who attempted suicide. Dimensions of attitude in the questionnaire include positivism, acceptability and religiosity, which were considered along with professional role and care, manipulation, personality traits, mental illness and discrimination.^26,27^

5.Attitudinal Beliefs Towards Suicidal Behavior Scale (CCCS-18):

The CCCS-18 consists of items that measure respondents’ beliefs about legitimacy of suicide (as a rationally acceptable act), acceptability of suicide in terminal patients, morality of suicide from a social perspective and suicide itself as a solution to exit from a given situation.^2^

6.Chinese Attitude Towards Suicide Questionnaire (CASQ-HK):

This scale was built up of three parts.^28^ Part A is composed of 73 statements about attitudes toward suicide. Part B includes 12 statements about 12 difficult scenarios. Part C contains 13 items about socio demographic characteristics, presence of serious suicidal ideation before suicidal attempt and knowing someone who attempted or committed suicide previously.^29^

7.Suicide Behavior Attitude Questionnaire (SBAQ):

The SBAQ is a self-administered instrument comprising 21 attitude statements that reflect clinical situations regularly experienced by healthcare personnel followed by a visual analogue scale to assess their beliefs and attitudes toward suicide attempters. The questionnaire has cognitive, affective and behavioral components. It must be completed by nursing personnel.^4,30^

8.Suicide Attitude Vignette Experience Scale (SAVE):

This scale consists of hypothetical scenarios in which the scheme’s main character experiences a problem and attempts suicide. The scale wants respondents to determine the extent to which they have sympathy or empathy with the character and agree with his/her decision in attempting suicide.^31^

9.Semantic Differential Scale Attitudes towards Suicidal behavior (SEDAS ):

This scale that includes 15 items was devised to measure attitudes towards suicidal behavior of different actors in various situations. Health/illness and acceptance/rejection are two dimensions that are used to score respondents attitudes. The semantic differential rating scale was planned to ask respondents to choose their position regarding attitude toward suicide between two bipolar conditions. Therefore; this scale has the property of being able to measure both intensity and direction of attitude in population surveys.^32^

10.Suicide Attitude Questionnaire (SUIATT):

The instrument is a 63-item questionnaire. This questionnaire asks about attitude and opinions toward self-destructive behavior, including thoughts about circumstances under which someone might attempt or commit suicide. This instrument is intended to measure attitudes of the respondents toward suicide committed by strangers, loved ones, and social groups under certain circumstances.^28,33^ The scale is proposed to be applicable across communities in comparative studies of attitudes toward suicide.

11.Attitude Towards Suicide Scale (ATSS):

The Attitude towards Suicide Scale (ATTS) is a relatively short questionnaire to examine the respondents’ opinions and attitudes towards suicide that includes 24 items classified in the 6 groups of factors.^34-36^ Acceptability of suicide, consideration of suicide as a sign of mental illness, believing in punishment of suicide committers after this life, necessity of communicating suicidal problems, indispensability of hiding suicidal behavior in the family and open reporting and discussion of suicide are the intended components in the scale.

12.Suicide Attitudes and Attribution Scale (SAAS):

In this scale, an assumed suicide case description from three predetermined scenarios is presented and respondents are requested to indicate their degree of agreement after reading 36 statements about the suicidal act and characteristics of suicide committer in the provided fiction through marking a number on a Likert type seven point scale.^37^ These statements were sorted in one of the six factors that represent negative view of the respondents towards suicide and suicide committer, his/her positive attitude towards suicide, belief in relatedness of the suicide to the personality of the attempters or to the external factors and considering suicide as a resolute and having a real wish to die.

13.General Social Survey's four questions (GSS 4):

The General Social Survey (GSS) is a national countrywide sociological survey in the United States that is conducting by the National Opinion Research Center (NORC) at the University of Chicago since 1972 to collect data about attitudes of the residents towards a wide range of topics including suicide. The scale consist of four questions on suicidal attitudes that ask the respondents their opinions about justifiability of committing suicide in each of the four life crises i.e. confronting incurable disease and bankruptcy or dishonored his/her family and being tired of living.^38^

14.Scale of Public Attitudes about Suicide (SPAS):

This scale was developed in China to measure attitudes towards suicide in the Chinese context. The questionnaire consists of 47 items, which are divided in into seven subscales (44 items), and 3 items that assess knowledge of the respondents about suicide. The subscales cover attitudes about preventability of suicide, ability to control tendency toward suicide, approving or disapproval of suicide, having empathy with the suicide committer, objective(s) of suicidal act, considering suicide as a social problem and differences between suicide attempt and successful suicidal act.^30^

Among the identified measures, 21 instruments were also suggested to be applicable for assessing suicidal ideation as embodied in Table 2.

1.Paykel’s questionnaire

Paykel’s questionnaire consists of five questions about suicidal thoughts and attempts, including:

Life-weariness, death wishes, suicidal ideation, suicidal plans and suicide attempts.^39-42^

2.Suicidal Ideation Questionnaire (SIQ)

This scale is a self-report measure of suicidal ideation that includes 30 items to assess specific thoughts and cognitions about suicide and death over the past month. Higher scores in the SIQ scale represent greater severity of suicidal ideation.^43^ Another version of this scale (SIQ-JR) that consists 15 items was prepared for evaluation of severity and frequency of suicidal ideation amongst junior high school students.^44,45^ Respondents are asked to rank their status on a 7 point grading scale that ranges from 0-6 to indicate never having suicidal taughts to almost having these thaughts everyday. Sum of individual item scores will reflect severity of suicidal ideation in the target group.

3.Adulte Suicidal Ideation Questionnaire (ASIQ)

This 25 items self-report inventory was adapted to measure current level of suicidal ideation in adults. Based on the attained information the respondents’ thoughts about suicide within the past month and his/her mental health could be apprehended.^46-49^ The items are rated on a 7-point grading system and the respondents’ total score should be compared to the provided cut-off points in the built in scoring key in order to recognize those in need of further scrutiny for suicide risk.

4.Suicidal Behavior Questionnaire (SBQ)

The Suicidal Behavior Questionnaire is a 30-item (previously 90-item) self-report measure of past and future suicidal ideation, past suicide threats, likelihood of future attempts and risk for death by suicide. A revised version of the scale (SBQ-R) to assess 14 suicidal behaviors (SBQ-14) in the past several days including today, the last month, last 4 months, the last year and over a lifetime was also reconciled. The respondents’ behaviors are scored using a weighted summary score across each time interval. Nine additional items were also presumed to assess the severity of lifetime suicidal behavior, current suicide plan, availability of a method, social deterrents, attitudes towards suicide behavior and distress tolerance. A brief 4 items SBQ-R is also exist to measure past suicidal thoughts and attempts as the predictor of one’s future suicidal behavior.^50,51^

5.Suicide Ideation Scale (SIS)

This scale was designed for college students and consists of 10 suicidal ideas to measure suicidal ideation in the past year. The items can be scored from 1 to 5, and therefore total scores could range from 10 to 50. The respondents should be asked to keep in their minds the worst point in their life when answering the questions.^52,53^

6.Suicidal Ideation Screening Questionnaire (SIS-Q)

The 4 items interviewer administered scale was created to measure retrospectively the respondent suicidal ideation in the past 12 months through asking about sleep disturbance, mood disturbance, guilt and hopelessness.^54^

7.Beck Scale for Suicide Ideation (BSSI)

The BSSI consists of 19 items to identify the presence of suicide ideation and if identified its severity in the respondents.^55^ The items can be used to assess any suicidal plans, deterrents to suicide and the extent of the respondents’ willingness to disclose his/her suicidal thoughts. This scale was modeled based on the interviewer rated version of the questionnaire by Beck et al.^56^ In some studies^57^ it was recommended to use the first five items as the screener and if a respondent score was 0 on items 4 and 5, the patient should be directed to item 20. The score range could be from 0-38 and a higher score indicates higher level of suicidal ideation.

8.Modified Scale for Suicide Ideation (MSSI)

The scale, which is a revised version of the BSSI, was created to assess the presence or absence of suicide ideation in the previous 48 hours and the degree of its severity.^58^The MSSI is a self-report measure and has 18 items and each item could be scored from 0-3. A total score of equal or higher than 21 indicates sever suicidal ideation of the respondent. The scale’s items were derived from the BSSI and include 5 additional items to measure intensity of ideation, courage and competence to attempt, and any talk or writing about death. In the MSSI, the first 4 items have been designated as screening items to identify the cases with severe suicidal ideation that necessitate whole scale administration.^59^

9.Suicide Probability Scale (SPS)

This scale was developed to help practitioners in measuring the risk of suicidal behavior. The SPS is a 36-item, self-report measure of attitudes and behaviors related to suicide risk including a sub scale that measures suicidal ideation specifically. The questionnaire could be applicable in inpatient, outpatient as well as general population. The items must be answered under the supervision of a trained professional. Based on a 4-point scale the respondents are requested to determine how often each statement in the items applies to them. Four sub scales are also proposed to provide in depth information about the respondents’ hopelessness, hostility, negative self-evaluation and suicide ideation.^51,60,61^

10.Positive and Negative Suicide Ideation (PANSI)

PANSI is a widely used self-report instrument to assess suicide related positive and negative thoughts (6 items; PANSI-PI and 8 items; PANSI-NSI). Respondents are requested to rate each item based on their thoughts during the past two weeks using a 5-point Likert scale, ranging from 1 (“none of the time”) to 5 (“most of the time”).^62^

11.Suicidal Behaviors Questionnaire—Revised (SBQ-R)

SBQ-R is a 4-item scale that focuses on the history of suicide-related behaviors and intentionality amongst the respondents.^63^

The item 1 refers to lifetime suicide ideation or attempt, item 2 to the frequency of suicidal ideation over the past 12 months, item 3 assesses the threat of suicide and item 4 the likelihood of suicidal behavior in the future.

12.InterSePT Scale for Suicidal Thinking (ISST)

Is a 12-item instrument for the assessment of current suicidal ideation in patients with schizophrenia and schizoaffective disorders.^64^ The scale was derived from the BSSI by deleting redundant items and could be administered by clinicians or researchers.

13.Geriatric Suicide Ideation Scale (GSIS)

The GSIS is a multi-dimensional measure of suicide-related ideation and associated factors developed for use with older adults. The GSIS is a 31-item, 5 point Likert-scale questionnaire and has 4 focuses areas: suicidal ideation (10 items), perceived life orientation (8 items), loss of personal and social worth (7 items) and death ideation (5 items).^65,66^

14.Columbia Suicide Severity Rating Scale (C-SSRS)

The C-SSRS is a semi-structured assessment tool that can be administered by clinicians or researchers and meets the recommended guidance of FDA (US Food and Drug Administration) for prospective assessment of suicidal ideation and behavior^67^ but its psychometric standards are under scrutiny based on the recent research evidence.^68^ The scale could measure suicidal ideation and intensity, suicidal behaviors and their lethality. The C-SSRS demonstrated high sensitivity and specificity for suicidal behavior classifications compared with other suicidal behavior scales.^67^

15.Five-item Brief Symptom Rating Scale (BSRS-5)

The BSRS-5 is a self-administered questionnaire that contains five items of psychological symptoms and is commonly used for screening psychological disorders. The BSRS-5 is derived from the 50-item Brief Symptom Rating Scale.^69^ The respondents are requested to answer on a five-point Likert-type scale that ranges from 0-4 (0 represents not at all and 4 represents extremely) whether they had anxiety, depression, hostility, interpersonal sensitivity and also as an additional symptom had trouble falling asleep in the past week. Another question i.e. "Do you have any suicide ideation” was also added to the end of questionnaire.^70^ A total score above 14 or score of more than 1 on the last question of the scale may indicate a severe mood disorder.

## Discussion

Fourteen suicidal attitude scales and 15 scales for assessing suicidal ideation were identified in this systematic review. Internal consistency, stability and reproducibility of the scales were described in the included publications along with their validity and reliability in the original or secondary studies. The range of questions in the identified measures and their inherent variations are stemmed from broad range of attitudes that might exist towards suicide. Considering the variety of the identified measures to assess suicidal attitude and ideation, the nature of suicidal attitudes and cross-cultural ambiguities in the approaches people may have about suicide itself or its contributing factors should be scrutinized carefully in choosing a measure for research purposes. Due to the implicit instability of attitudes, the scales validity might be deteriorate over time therefore; identified tools may not be reliable to be applied for diagnostic or research purposes even if their validity has been approved in previous studies. Diverse considered dimensions of attitudes in the identified scales exhibit the lack of required consensus about these dimensions. Such a disagreement was also emphasized in previous studies.^7,13-16^

In addition to the complications posed by the structure of the identified scales, feasibility of the scales’ application in the clinical or research settings should be examined based on the number of items each of the scales have and their rating complexities in practice. A robust validity of the translated versions of the scales in the local cultural settings is another important factor that should be ascertained. The range of population(s) for which these scales were designed and the main objectives of the scales developer were important aspects that needs to be investigated.

Inclusion of publications written in English and the limited number of databases searched for relevant research evidence may have caused selection and accessibility bias in this review. However, the authors do not believe that major psychometrically approved scales were out-listed.

## Conclusion

Based on the observed diversities in the identified scales it can be concluded that there is no gold standard approach to study suicide related attitudes and ideations. The overall recommendation for scholars is to focus on generally agreed dimensions of suicidal ideation and attitudes and cross-cultural validation of the introduced scales in order to be applicable in different ethnic and socially diverse populations.

## Acknowledgements

This study was financed by a grant provided by the Tabriz University of Sciences (5/53/4330) and approved by the Tabriz University of Medical Sciences Review Board for Ethics in Research on Humans. This paper was prepared based on parts of the findings of a research completed to fulfill the first author’s requirements for the MSPH thesis in the department of Health Education & Promotion at the Tabriz University of Medical Sciences.

## Competing Interests

The authors declare that there is no conflict of interests regarding the publication of this paper.

## References

[1] Norheim AB, Grimholt TK, Ekeberg Ø (2013). Attitudes towards suicidal behaviour in outpatient clinics among mental health professionals in Oslo. BMC Psychiatry.

[2] Carmona-Navarro MC, Pichardo-Martinez MC (2012). Attitudes of nursing professionals towards suicidal behavior: influence of emotional intelligence. Rev Lat Am Enfermagem.

[3] Eskin M, Voracek M, Stieger S, Altinyazar V (2011). A cross-cultural investigation of suicidal behavior and attitudes in Austrian and Turkish medical students. Soc Psychiatry Psychiatr Epidemiol.

[4] Berlim MT, Perizzolo J, Lejderman F, Fleck MP, Joiner TE (2007). Does a brief training on suicide prevention among general hospital personnel impact their baseline attitudes towards suicidal behavior?. Affect Disord.

[5] Cashin MA. Analyses of the structure and preliminary psychometric properties of the Multi-Attitude Suicide Tendency Scale-II (MAST-II) [Master Thesis]. The University of Texas at San Antonio;2012.

[6] Lee JI, Lee MB, Liao SC, Chang CM, Sung SC, Chiang HC (2010). Prevalence of suicidal ideation and associated risk factors in the general population. J Formos Med Assoc.

[7] Goldston DB. Assessment of suicidal behaviors and risk among children and adolescents [Internet]. National Institute of Mental Health; 2000. [cited 2014 Aug 10]. Available from: http://www.sprc.org/sites/sprc.org/files/library/Goldston Assessment Suicidal Behaviors Risk Children Adolescents.pdf.

[8] Baca-Garcia E, Perez-Rodriguez MM, Mann JJ, Oquendo MA (2008). Suicidal behavior in young women. Psychiatr Clin North Am.

[9] Vanderoost F, van der Wielen S, van Nunen K, Van Hal G (2013). Employment loss during economic crisis and suicidal thoughts in Belgium: a survey in general practice. Br J Gen Pract.

[10] Corson K, Denneson LM, Bair MJ, Helmer DA, Goulet JL, Dobscha SK (2013). Prevalence and correlates of suicidal ideation among Operation Enduring Freedom and Operation Iraqi Freedom veterans. J Affect Disord.

[11] Mofidi N, Ghazinour M, Salander-Renberg E, Richter J (2008). Attitudes towards suicide among Kurdish people in Iran. Soc Psychiatry Psychiatr Epidemiol.

[12] Fazakas-DeHoog LL. An integrated cognitive-affective model of suicidal thinking and behavior [Ph.D Thesis]. Canada: The University of Western Ontario;2008.

[13] Kodaka M, Postuvan V, Inagaki M, Yamada M (2011). A systematic review of scales that measure attitudes toward suicide. Int J Soc Psychiatry.

[14] Hourani LL, Jones D, Kennedy K, Hirsch K. Review article: update on suicide assessment instruments and methodologies. San Diego, CA: Washington, DC: Naval Health Research Center; Bureau of Medicine and Surgery;1999. Report No. 99-31.

[15] Brown GK. A review of suicide assessment measures for intervention research with adults and older adults. Philadelphia: National Institute of Mental Health; 2001.

[16] Anderson M, Standen P, Nazir  S, Noon  JP (2000). Nurses’ and doctors’ attitudes towards suicidal behaviour in young people. Int J Nurs Stud.

[17] Domino G, Moore D, Westlake L, Gibson L (1982). Attitudes toward suicide: A factor analytic approach. J Clin Psychol.

[18] Domino G (1996). Test-retest reliability of the Suicide Opinion Questionnaire. Psychol Rep.

[19] Orbach I, Milstein I, Har-Even D, Apter A, Tiano S, Elizur A (1991). A Multi-Attitude Suicide Tendency Scale for adolescents. J Consult Clin Psych.

[20] Floor J. A graduate level course on adolescent suicide: Addressing assessment, intervention, and postvention [Psy.D Dissertation]. The Chicago School of Professional Psychology;2010.

[21] Omma L, Sandlund M, Jacobsson L. Suicidal ex-pressions in young Swedish Sami, a cross-sectional study. Int J Circumpolar Health 2013;72. doi: 10.3402/ijch.v72i0.19862 10.3402/ijch.v72i0.19862 PMC3549065 23346555

[22] Renberg ES, Hjelmeland H, Koposov R (2008). Building models for the relationship between attitudes toward suicide and suicidal behavior: based on data from general population surveys in Sweden, Norway, and Russia. Suicide Life Threat Behav.

[23] Rodríguez AH, Caldera T, Kullgren G, Renberg ES (2006). Suicidal expressions among young people in Nicaragua: a community-based study. Soc Psychiatry Psychiatr Epidemiol.

[24] Renberg ES, Jacobsson L (2003). Development of a questionnaire on attitudes towards suicide (ATTS) and its application in a Swedish population. Suicide Life Threat Behav.

[25] Gawley KK. Information seeking by survivors of suicide: What did they know, when did they know it, how did they find it, and was it helpful? [Psy.D Dissertation]. Rutgers The State University of New Jersey, Graduate School of Applied and Professional Psychology;2010.

[26] Neville K, Roan NM (2013). Suicide in hospitalized medical-surgical patients: exploring nurses' attitudes. J Psychosoc Nurs Ment Health Serv.

[27] Ouzouni C, Nakakis K (2009). Attitudes towards attempted suicide: the development of a measurement tool. Health Sci J.

[28] Jiao Y, Phillips MR, Sheng Y, Wu G, Li X, Xiong W (2014). Cross-sectional study of attitudes about suicide among psychiatrists in Shanghai. BMC Psychiatry.

[29] Lee S, Tsang A, Li XY, Phillips MR, Kleinman A (2007). Attitudes toward suicide among Chinese people in Hong Kong. Suicide Life Threat Behav.

[30] Botega NJ, Reginato DG, da Silva SV, Cais CF, Rapeli CB, Mauro ML (2005). Nursing personnel attitudes towards suicide: the development of a measure scale. Rev Bras Psiquiatr.

[31] Smith CE. Attitude toward suicidal women based on gender of the participant and race of the target figure [Master Thesis]. Tennessee: East Tennessee State University;2001.

[32] Jenner JA, Niesing J (2000). The construction of the SEDAS: a new suicide-attitude questionnaire. Acta Psychiatr Scand.

[33] Ramos O. A leadership perspective for understanding police suicide: An analysis based on the Suicide Attitude Questionnaire [Ph.D Thesis]. Capella University;2007.

[34] Eskin M (2004). The effects of religious versus secular education on suicide ideation and suicidal attitudes in adolescents in Turkey. Soc Psychiatry Psychiatr Epidemiol.

[35] Nader IW, Tran US, Baranyai P, Voracek M (2012). Investigating dimensionality of Eskin's attitudes toward suicide scale with Mokken scaling and confirmatory factor analysis. Arch Suicide Res.

[36] Sorjonen K (2005). Attitudes toward suicide as a function of the victim's physical status. Omega - J Death Dying.

[37] Zhang J, Jia CX (2009-2010). Attitudes toward suicide: the effect of suicide death in the family. Omega (Westport).

[38] Hem E, GrLnvold NT, Aasland OG, Ekeberg O (2000). Hem E, GrLnvold NT, Aasland OG, Ekeberg OThe prevalence of suicidal ideation and suicidal attempts among Norwegian physiciansResults from a cross-sectional survey of a nationwide sample. Eur Psychiatry.

[39] Bartels SJ, Coakley EH, Oxman TE, Constantino G, Oslin D, Chen H (2002). Suicidal and death ideation in older primary care patients with depression, anxiety, and at-risk alcohol use. Am J Geriatr Psychiatry.

[40] Nazem S, Siderowf AD, Duda JE, Brown GK, Ten Have T, Stern MB (2008). Suicidal and death ideation in Parkinson's disease. Mov Disord.

[41] Sterud T, Hem E, Lau B, Ekeberg O (2008). Suicidal ideation and suicide attempts in a nationwide sample of operational Norwegian ambulance personnel. J Occup Health.

[42] Ghazinour M, Mofidi N, Richter J (2010). Continuity from suicidal ideations to suicide attempts? An investigation in 18-55 years old adult Iranian Kurds. Soc Psychiatry Psychiatr Epidemiol.

[43] Stanley IH, Snyder DJ, Westen S, Ballard ED, Teach SJ, Kapetanovic S (2013). Self-Reported Recent Life Stressors and Risk of Suicide in Pediatric Emergency Department Patients. Clin Pediatr Emerg Med.

[44] Lugo-Stalker MI. Cross-cultural differences in coping styles in juvenile offenders [Psy.D Dissrtation]. University of Hartford;2002.

[45] Steyn R, Vawda N, Wyatt GE, Williams JK, Madu SN (2013). Posttraumatic stress disorder diagnostic criteria and suicidal ideation in a South African Police sample. Afr J Psychiatry (Johannesbg).

[46] Emmerich A. The Multidimensional Suicide Inventory-28 (MSI-28): Evaluation of factor structure and psychometric properties [Master Thesis]. The University of Texas at San Antonio;2012.

[47] Kuramoto SJ. Suicidal ideation among inner-city drug users: Association with social support networks and polydrug use [Ph.D Dissertation]. The Johns Hopkins University;2011.

[48] Ranjan JK, Jahan M, Singh AR, Singh DK, Majhi G (2011). Suicidal ideation among chronic schizophrenic patients. J Behav Sci.

[49] Simon NM, Zalta AK, Otto MW, Ostacher MJ, Fischmann D, Chow CW (2007). The association of comorbid anxiety disorders with suicide attempts and suicidal ideation in outpatients with bipolar disorder. J Psychiatr Res.

[50] Watkins RL. Exposure to peer suicide in college students [Ph.D Dissertation]. Northern Illinois University;2004.

[51] Hsu EM. A validation study of the cultural assessment of suicide risk among Latino/a American adults [Ph.D Dissertation]. Palo Alto University;2013.

[52] Wallack CE. Factors associated with suicidal ideation among American college students: A re-examination of the escape theory of suicide [Ph.D Dissertation]. University of Florida;2007.

[53] Yang MS, Yang MJ (2000). Correlated risk factors for suicidal ideation in aboriginal Southern Taiwanese women of childbearing age. Public Health.

[54] Coleman H. The relationships between suicidal ideation and three aspects of the self in 18- and 19-year-old college students [Ph.D Dissertation]. Alliant International University, San Francisco Bay;2007.

[55] Purcell B, Heisel MJ, Speice J, Franus N, Conwell Y, Duberstein PR (2012). Family connectedness moderates the association between living alone and suicide ideation in a clinical sample of adults 50 years and older. Am J Geriatr Psychiatry.

[56] Nichols E. Variations in suicidal ideation among substance users [Master Thesis]. University of North Texas;2012.

[57] O’Connor E, Gaynes BN, Burda BU, Williams C, Whitlock EP. Screening for Suicide Risk: A systematic evidence review for the U.S. preventive services task force [Internet]. Rockville (MD): Agency for Healthcare Research and Quality (US); 2013 Apr. Report No.: 13-05188-EF-1. [cited 2014 June 18]. http://www.ahrq.gov/downloads/pub/prevent/pdfser/suicidser.pdf. 23678511

[58] Beck AT, Steer RA, Rantieri WF (1988). Scale for suicide ideation: psychometric properties of a self-report version. J Clin Psychol.

[59] Beck AT, Kovacs M, Weissman A (1979). Assessment of suicidal intention: the scale of suicide ideation. J Consult Clin Psychol.

[60] van Spijker BA, van Straten A, Kerkhof AJ (2010). The effectiveness of a web-based self-help intervention to reduce suicidal thoughts: a randomized controlled trial. Trials.

[61] Miller IW, Norman WH, Bishop SB, Dow MG (1986). The Modified Scale for Suicidal Ideation: reliability and validity. J Consult Clin Psychol.

[62] Osman A, Gutierrez PM, Jiandani J, Kopper BA, Barrios FX, Linden SC (2003). A preliminary validation of the Positive and Negative Suicide Ideation (PANSI) inventory with normal adolescent samples. J Clin Psychol.

[63] Osman A, Bagge CL, Gutierrez PM, Konick LC, Kopper BA, Barrios FX (2001). The Suicidal Behaviors Questionnaire-Revised (SBQ-R): validation with clinical and nonclinical samples. Assessment.

[64] Lindenmayer JP, Czobor P, Alphs L, Nathan AM, Anand R, Islam Z (2003). The InterSePT scale for suicidal thinking reliability and validity. Schizophr Res.

[65] Heisel MJ, Flett GL (2006). The development and initial validation of the geriatric suicide ideation scale. Am J Geriatr Psychiatry.

[66] Heisel  MJ, Flett GL, Besser A (2002). Cognitive functioning and geriatric suicide ideation: testing a mediational model. Am J Geriatr Psychiatry.

[67] Posner K, Brown GK, Stanley B, Brent DA, Yershova KV, Oquendo MA (2011). The Columbia–Suicide Severity Rating Scale: initial validity and internal consistency findings from three multisite studies with adolescents and adults. Am J Psychiatry.

[68] Giddens JM, Sheehan KH, Sheehan DV (2014). The Columbia-Suicide Severity Rating Scale (C–SSRS): Has the ―Gold Standard‖ Become a Liability?. Innov Clin Neurosci.

[69] Lee MB, Lee YJ, Yen LL, Lin MH, Lue BH (1990). Reliability and validity of using a Brief Psychiatric Symptom Rating Scale in clinical practice. J Formos Med Assoc.

[70] Lung FW, Lee MB (2008). The five-item Brief-Symptom Rating Scale as a suicide ideation screening instrument for psychiatric inpatients and community residents. BMC Psychiatry.

